# THE LONG LIVES OF PRIMATES AND THE ‘INVARIANT RATE OF AGING’ HYPOTHESIS

**DOI:** 10.1093/geroni/igad104.0197

**Published:** 2023-12-21

**Authors:** Susan Alberts

**Affiliations:** Duke University, Durham, North Carolina, United States

## Abstract

Can we slow the rate of human aging? A decades-long trend toward increasing life expectancy and greater lifespan equality in human populations raises the possibility that we can. But the rate of aging is closely correlated with other traits and may be highly constrained or even relatively fixed within species, according to the ‘invariant rate of aging’ hypothesis. To gain insight into biological constraints on aging, we use an unprecedented collection of datasets from 39 human and nonhuman primate populations, representing 7 genera distributed across the order Primates. We show that the highly regular linear relationship between life expectancy and lifespan equality reported in humans is recapitulated in other primate genera, and that variation in the rate of aging is several orders of magnitude smaller than pre-adult and age-independent mortality, which are highly variable within genera. Thus, within primate genera, longer life expectancies are associated with fewer early deaths, but not with a lower rate of aging. We also demonstrate that changes in the rate of aging, but not other ageing parameters, can produce striking, species-atypical changes in mortality patterns. Our results support the invariant rate of aging hypothesis, suggesting biological constraints on how much we can slow the human rate of aging.

